# Comparing the Effects of Short-Term Liuzijue Exercise and Core Stability Training on Balance Function in Patients Recovering From Stroke: A Pilot Randomized Controlled Trial

**DOI:** 10.3389/fneur.2022.748754

**Published:** 2022-02-10

**Authors:** Ying Zhang, Chen Wang, JianZhong Yang, Lei Qiao, Ying Xu, Long Yu, Jie Wang, Weidong Ni, Yan Wang, Yue Yao, ZhiJie Yong, ShanShan Ding

**Affiliations:** Department of Rehabilitation, Shanghai Xuhui Central Hospital, Zhongshan-Xuhui Hospital, Fudan University, Shanghai, China

**Keywords:** short-term Liuzijue Qigong, balance functions, core stability training, pilot randomized controlled trial, stroke

## Abstract

**Aims:**

Liuzijue Qigong (LQG) exercise is a traditional Chinese exercise method in which breathing and pronunciation are combined with movement guidance. Breathing is closely related to balance, and LQG, as a special breathing exercise, can be applied to balance dysfunction after stroke. The purpose of this study was to observe the clinical effects of short-term LQG exercise on balance function in patients recovering from stroke.

**Methods:**

Stroke patients were randomly divided into an Intervention Group (IG) (*n* = 80) and a Control Group (CG) (*n* = 80). The IG received conventional rehabilitation training plus LQG and the CG received conventional rehabilitation training plus Core Stability Training (CST). All patients received treatment once a day, 5 times a week for 2 weeks. The primary outcome was Berg Balance Scale (BBS). Secondary outcome measures were static standing and sitting balance with eyes open and closed, Fugl-Meyer Assessment (FMA), Maximum Phonation Time (MPT), Modified Barthel Index (MBI) and diaphragm thickness and mobility during quiet breath (QB) and deep breath (DB).

**Results:**

Compared with the CG, the IG showed significant improvement in the BBS (10.55 ± 3.78 vs. 9.06 ± 4.50, *P* = 0.039), MPT (5.41 ± 4.70 vs. 5.89 ± 5.24, *P* = 0.001), MBI (12.88 ± 6.45 vs. 10.00 ± 4.84, *P* = 0.003), diaphragmatic mobility during QB (0.54 ± 0.73 vs. 0.33 ± 0.40, *P* = 0.01) and diaphragmatic mobility during DB (0.99 ± 1.32 vs. 0.52 ± 0.77, *P* = 0.003), Cop trajectory in the standing position with eyes open (−108.34 ± 108.60 vs. −89.00 ± 140.11, *P* = 0.034) and Cop area in the standing positions with eyes open (−143.79 ± 431.55 vs. −93.29 ± 223.15, *P* = 0.015), Cop trajectory in the seating position with eyes open (−19.95 ± 23.35 vs. −12.83 ± 26.64, *P* = 0.001) and Cop area in the seating position with eyes open (−15.83 ± 9.61 vs. −11.29 ± 9.17, *P* = 0.002).

**Conclusions:**

The short-term LQG combined with conventional rehabilitation training significantly improved the balance functions of stroke patients. It also improved static standing and sitting balance with the eyes open, diaphragm functions, maximum phonation time and the quality of daily life for stroke patients.

**Clinical Trial Registration:**

http://www.chictr.org.cn/edit.aspx?pid=25313&htm=4, Identifier: ChiCTR1800014864.

## Introduction

It has previously been reported that balance dysfunction in stroke patients is caused by insufficient trunk muscle strength (1), which is often manifested as an inability to maintain balance and asymmetrical posture in a sitting or upright position, producing a poor ability to control the trunk while transferring and walking (2). Thus, the key to improving balance ability is to achieve effective core stability. The core skeletal muscle groups are composed of superficial and deep muscles, among which deep core muscles play an important role in maintaining trunk stability. It has been demonstrated that compared with the superficial core muscle group, the decline of the deep core muscle group is particularly severe in patients with stroke hemiplegia with their importance being their vital roles in maintaining balance (3).

Core functional training is one of the most widely used balance training methods in clinical practice and includes tummy tuck and hip bridge exercises that are more likely to activate superficial core rather than deep muscles (4). During the training, centripetal and centrifugal contractions with fast speed and high load intensity are performed, which do not effectively activate the deep core muscle groups. On the contrary, it leads to the aggravation of spasm in stroke patients (5). Therefore, we need to find a rehabilitation method that can effectively activate the deep core muscles of stroke patients and avoid aggravating the spasticity of the superficial core muscles, thus improving the balance functions of these patients.

Remarkably, the deep core muscles are also the main respiratory muscles, such as the diaphragm, multifidus and pelvic floor muscles. We generally found that the decline of the deep core muscle group in stroke patients lead to the decline of maximum inspiratory and maximum expiratory pressures (6, 7). A cross-sectional study found that maximum expiratory and maximum inspiration pressures were positively associated with static and dynamic balance (8, 9). This finding indicates that respiratory muscle function is closely related to balance functions after stroke (9). Studies have confirmed that respiratory function is critical to the improvement of balance functions in stroke patients (10, 11). Thus, breathing training is one of the important means of facilitating balance.

LQG is a traditional Chinese method based on breathing and combined with movement guidance (12), that is effective in improving lung ventilation in stroke and chronic obstructive pulmonary disease (COPD) patients (13, 14). For LQG, it adjusts the ups and downs of breathing by practicing the synchronized pronunciation of “Xu,” “He,” “Hu,” “Si,” “Chui” and “Xi,” together with slow and gentle inhalation and exhalation. These exercises effectively balance the energy and functions of the internal organs and play an important regulatory role for the liver, heart, spleen, lungs, kidneys and triple jiao (15, 16). The LQG is a special method of breathing training. We hypothesized whether it was possible to activate the respiratory muscle group through LQG, thereby activating the deep core muscle groups and benefiting balance functions. Therefore, the aim of the present study was to evaluate the effects of short-term LQG compared to CST on balance dysfunction in stroke patients.

## Methods

The study was approved and directed by the Ethics Committee of Shanghai Xuhui Central Hospital (No. 2017-40). It was a single-center, assessor-blind, randomized controlled trial registered on the website of the Chinese Clinical Trial Registry (identifier: ChiCTR1800014864). Before the trial began, all assessment and intervention personnel received uniform training and developed common operating standards. All evaluation and intervention procedures were performed in strict accordance with the standardized methods. All subjects enrolled in the study signed an informed consent form.

### Design

**Figure 1:**
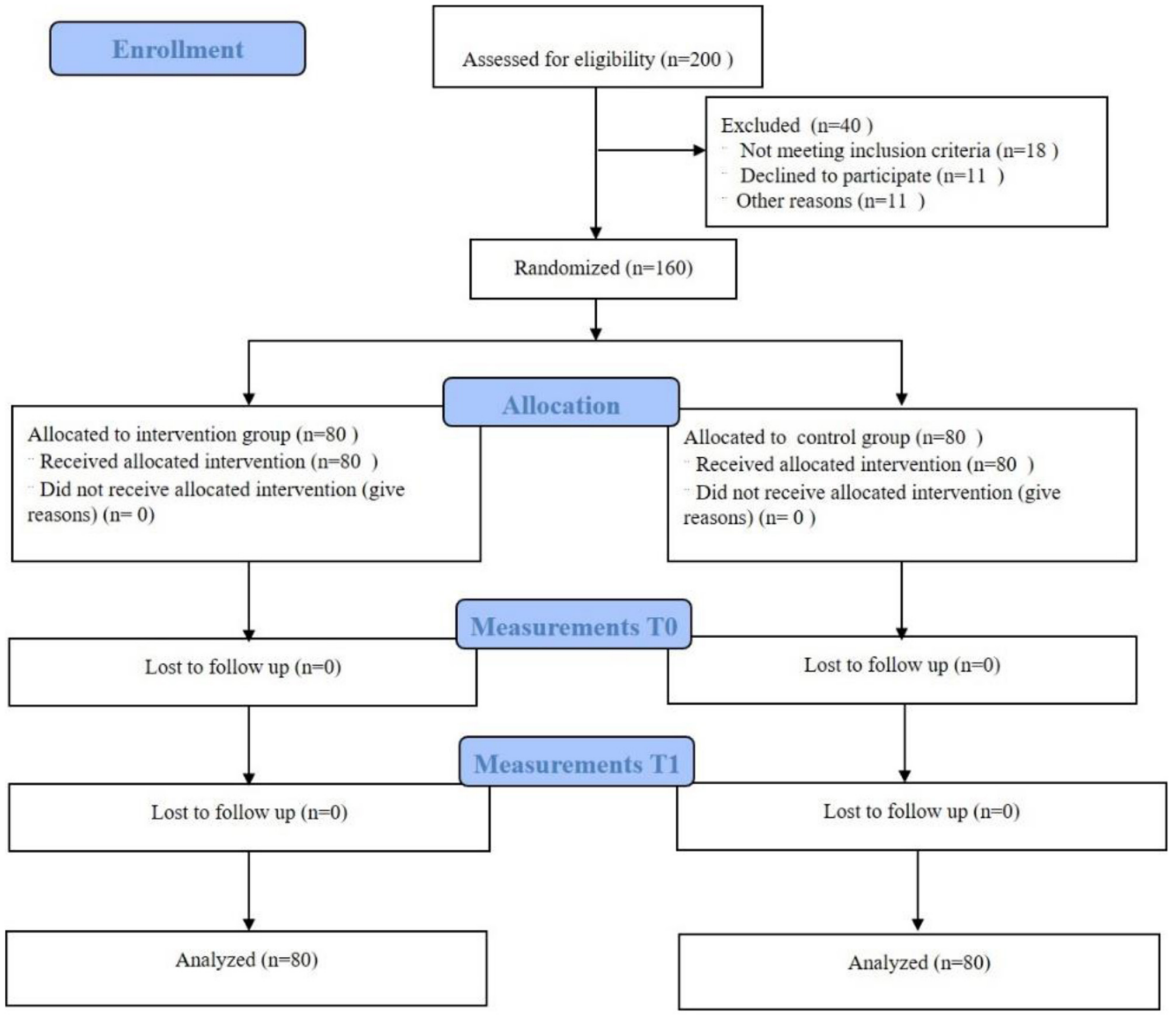
Flow chart of the pilot study.

The pilot study was a single-center, evaluator-blinded, randomized controlled trial (RCT) (Figure 1 gives an overview of the study design). This report includes the recommended elements elaborated in the pilot RCT reporting guidelines (Appendix 1: CONSORT checklist) (17).

### Setting

Shanghai is the second largest city in China and the most economically developed city. Located in East China, it has a resident population of 24.28 million. Shanghai Xuhui District Central Hospital is a tertiary public hospital, and is also a key specialist in cardiovascular and cerebrovascular rehabilitation of Shanghai Health Planning Commission and a key discipline construction unit of neurological rehabilitation of Shanghai Disabled Persons' Federation. It is the specialty of the Department of Chinese and Western medicine combined rehabilitation, and has created the clinical application of traditional qigong in stroke rehabilitation, which has achieved remarkable clinical results in the medical association units under its jurisdiction, which has laid the foundation for the smooth development of this study.

### Participants

The study recruited 160 eligible stroke patients from March 2018 to August 2019 in the rehabilitation department of Shanghai Xuhui Central Hospital. All subjects met the following inclusion criteria: (1) Diagnosis of cerebral hemorrhage or cerebral infarction (18); (2) Meet traditional Chinese medicine (TCM) diagnostic criteria of stroke (19); (3) Sitting balances > 2 levels; (4)Age range between 40 and 80 years; (5) The course of the disease was 2 weeks to 6 months; (6) People who were physically active and could tolerate 45 mins of exercise; (7) The patient was left with hemiplegia or quadriplegia, If the patient is quadriplegic after stroke, he or she should qualify for a Brunnstrum grade 4 or higher on at least one upper limb; (8) The patient's vital signs were stable; (9) Agreed to sign an informed consent form; (10) All subjects in the study were able to independently complete the static balance ability test.

Patients were excluded according to the following criteria: (1) the patient had no severe consciousness, cognitive dysfunction, aphasia or hemianopia; (2) patients with acute disease of the heart, brain, kidneys and other organs; (3) a patient had a severe psychological disorder and a Mini-Mental State Examination (MMSE) score ≤ 23.

### Schematic Process of the Pilot Study

The flow chart of this study is shown in Figure 1 and is consistent with the previously described study protocol.

### Randomization and Blinding

Based on a computer-generated table of random numbers, the researchers placed consecutive numbers into sealed envelopes and randomly assigned them in a 1:1 ratio.

And followed the guidelines for reporting trials comprehensive standards (CONSORT).

Only the principal investigator in this study will be aware of the order of random assignment. The assessor will be a trained external rehabilitation therapist. The assessor and the patient will not be able to communicate about the intervention during the assessment. In this study, only the assessor will be blinded and therefore the study will not involve unblinding.

### Intervention and Control

Patients in the intervention and control group received conventional rehabilitation training plus LQG or CST respectively. The duration of conventional rehabilitation training in the intervention and control groups was 30 min per session and the duration of treatment in the LQG (IG) and CST (CG) was 15 min for each patient. All patients received treatment once a day, 5 times a week, for a total of 2 weeks. Conventional rehabilitation training included manual therapy such as neurodevelopmental therapy, active and passive joint movements, balance and walking training, and occupational therapy, etc.

In CG, patients received 15 min of CST, besides convention rehabilitation training. CST included the following procedures (20, 21): (1) abdominal breathing training; in the supine position, the therapist placed their hand on the patient's abdomen, accompanied by rhythmic breathing for pressure or relaxation (4 min); (2) bridge movement; in the supine position, the subjects completed the supine curl, double bridge and single bridge, and the half bridge on the Swiss ball (6 min); (3) core control training; in the sitting position, the therapist controls the movement of the patient's pelvis in 4 directions, namely forwards, back, left, and right, for 10 sec accompanied by reaching of both upper limbs in each direction (5 min). The above exercises could be performed on both the stable plane (i.e., the treatment bed) and the unstable plane (i.e., sitting on a Swiss ball).

**Figure 2:**
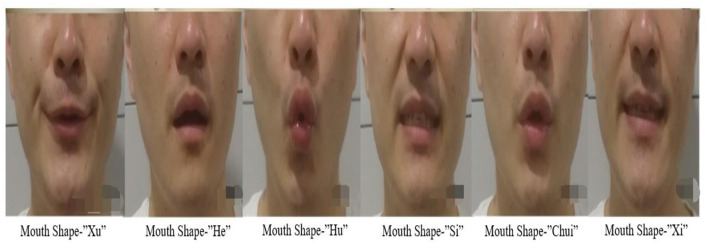
The mouth shape of LQG. It shows the pronunciation of the six characters of LQG: as follows: “Xu” and “Si” for the alveolar sound, “He” and “Xi” for the lingual sound, “Hu” and “Chui” for the labial sound.

Patients in the IG were required to receive 15 min of LQG in addition to completing regular rehabilitation training. The specific pronunciations were as follows (22): “Xu,” “He,” “Hu,” “Si,” “Chui” and “Xi,” above of the mouth patterns is shown in Figure 2.

- “Xu” is assisted by the teeth. The upper and lower teeth should be parallel, leaving a gap between the teeth and the tongue. Air is exhaled from the gaps, and the lips are pulled back slightly.
- “He” is assisted by the tongue; air is exhaled between the tongue and the roof of the mouth.
- “Hu” is assisted by the throat and round lips, and then the air is slowly exhaled.
- “Si” is assisted by teeth; the upper and lower teeth are parallel and there is a narrow gap between them. The tip of tongue lightly touches the lower teeth; the air is exhaled between the teeth.
- “Chui” is assisted by the lips. The tongue and the lips are pulled back so that the upper and lower teeth are parallel; then the air in the throat comes out through the sides of the tongue and between the stretched lips.
- “Xi” needs to be performed with the assistance of the teeth. The lower teeth must touch the tip of the tongue and the lips are slightly extended back. Air is exhaled through the back teeth (23, 24).

In addition to the above articulation points, different body movements should accompany each phonation in order to achieve deeper and longer breathing. Every word and action were repeated 6 times.

**Figure 3:**
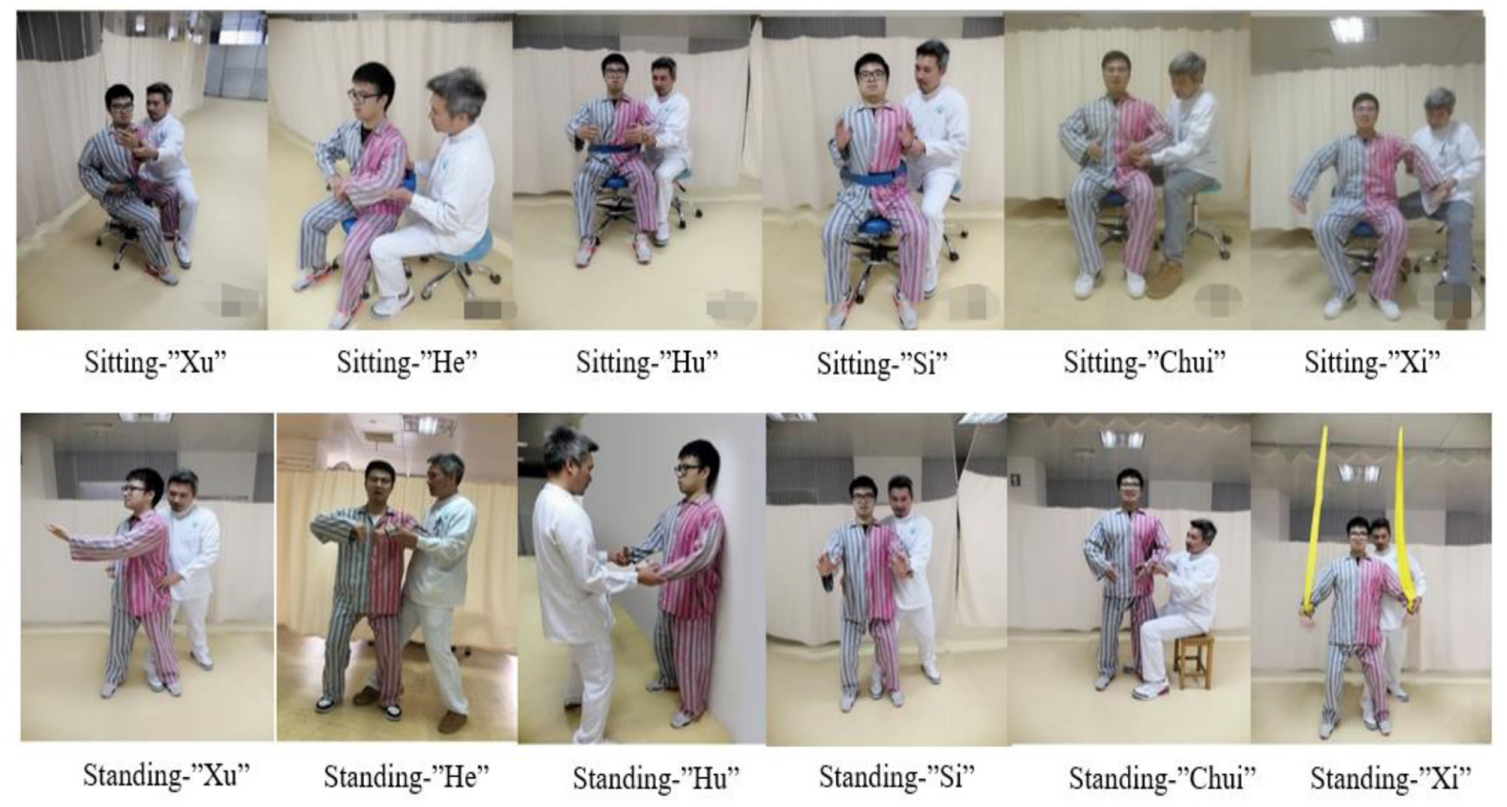
The application of LQG movements in stroke patients. The diagram shows the application of LQG movements in a stroke patient. The therapist uses LQG movements for the stroke patient in the sitting and standing positions. In sitting position, the patient is unable to perform the upper limb detachment independently and requires the therapist to assist him/her with the upper limb guide movements. In standing position, the therapist assists the patient with trunk rotation and upper limb movements, and controls the knee joint to move the center of gravity up and down. Assistive devices, such as elastic bands, may also be used to perform upper limb movements with resistance.

Stroke patients with hemiplegia often present with varying degrees of balance ability or limb functionality. LQG is difficult for them to perform accurately and independently without assistance. Therefore, the patients need to be trained in sitting or standing depending on their balance ability. For patients with upper limb Brunnstrom functional level below grade four, the physiotherapist should assist the hemiplegic limb to complete the movement and maintain bilateral symmetry. in the standing position, the therapist could use their own knee to support the patient's popliteal fossa on the affected side to assist in shifting the center of gravity up and down. The movements of LQG are in Figure 3.

### Data Collection

#### Baseline Data

The following baseline data are collected mainly through case report forms which include: (1) socio-demographic; (2) lesion location and duration; (3) collection of functional indicators.

#### Patient Adherence to the Intervention

The patient's compliance with the exercise is mainly recorded through a daily exercise log, including the length of each session, the number of sessions and the patient's physical tolerance, and the therapist is required to describe the specifics of the patient's intervention.

#### Clinical Outcome Measures From Both Groups

The balance function of stroke patients and healthy elderly people is usually assessed by the BBS. It has 14 items, each score is 0–4 points, and the total score is 56 points. The higher the score, the better the balance function, while the lower the score, the worse the balance function is (25).

The simplified FMA is often used in clinical rehabilitation for the assessment of motor function with better reliability and validity (26). The total score of 100 is divided into upper and lower limb motor function assessments, with the upper limb scoring 56 and the lower limb scoring 44 (27). Each item has a 3-point scale, with higher scores indicating better motor function. Their functional impairment is classified as follows. <50 = severe motor impairment; 50–84 = significant motor impairment; 85–95 = moderate motor impairment; 96–99 = mild motor impairment; and 100 = normal.

The assessment of patients' quality of daily life was completed by using the MBI, with a maximum score of 100 that assesses 10 items, each of which is given a score between 0 and 10 points. The higher the score, the better the ability of daily living for patients, while the lower the score the worse is their quality of daily life (28).

The duration of articulation of simple vowels after a deep breath will be measured using the MPT (29). The patient was measured using audio recording software in a mobile phone. The patient was asked to produce a sustained vowel /a:/ for as long as possible and was verbally encouraged in the process (30). The variability and reliability of the method for this measurement was good. Three consecutive trials were allowed with an interval of 15 secs between each trial (31). The measurement requirements of MPT are as follows: (1) the longer the pronunciation time, the better; (2) breathing evenly; (3) even breathing loudness; and (4) pitch within the correct frequency range.

At the same time, the relatively longer MPT value will be taken as the final evaluation result.

**Figure 4:**
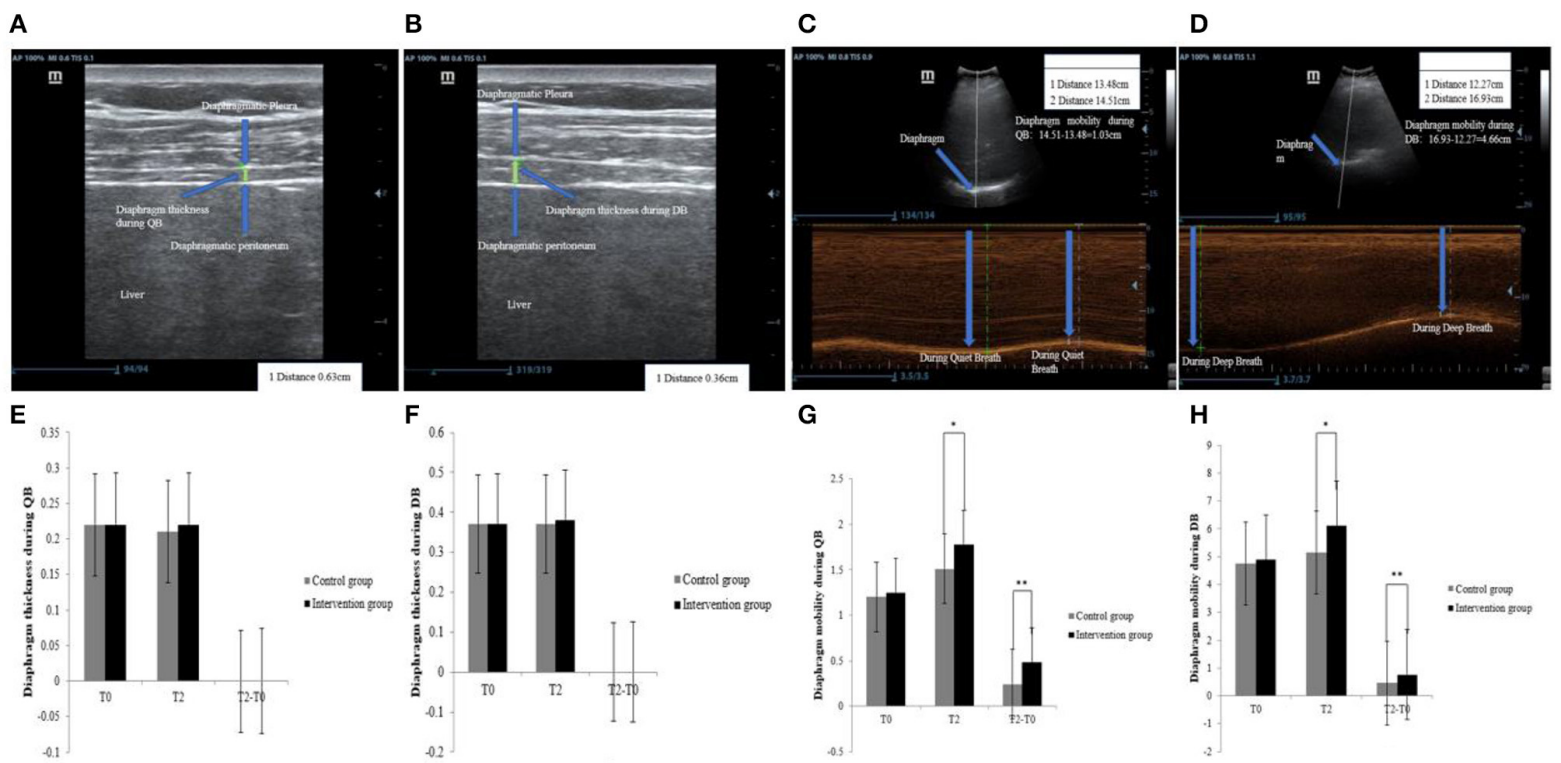
Ultrasound and comparison of diaphragm thickness and mobility. **(A)** diaphragm thickness during QB, **(B)** diaphragm thickness during DB, **(C)** diaphragm mobility during QB, **(D)** diaphragm mobility during DB, **(E)** comparison of diaphragm thickness during DB, **(F)** comparison of diaphragm mobility during QB, **(G)** comparison of diaphragm mobility during QB, **(H)** comparison of diaphragm mobility during DB. QB, Quiet Breath; DB, Deep Breath; *Significant differences between groups (*P* < 0.05); **The difference between groups was highly significant (*P* < 0.01).

A MyLab color Doppler ultrasound diagnostic instrument with a probe frequency of 5-10 MHz was used to determine the thickness of the diaphragm and its mobility. Because the ultrasound image of the right diaphragm is clearer than the left, the right side was selected for uniform measurement by a specially trained physician (32). Diaphragm thickness was measured during quiet breath and deep breath (Figure 4). The diaphragm thickening fraction (DTF) was calculated as: DTF = (end-inspiratory thickness—end-expiratory thickness) / end-expiratory thickness × 100% (33, 34). To measure the diaphragmatic movement, the caliper was placed on the baseline or the top of the diaphragmatic echo line while the patient was breathing quietly or taking deep breaths. The distance between the above 2 lines was measured in the frozen image (Figure 4). The average of 3 different cycles was calculated for statistical analysis (35–37).

A PK-254P dynamic and static balance meter was used to evaluate the patient's static balance ability (38). The aim was to assess the patient's static balance between the upright and seated positions in 2 different modes, namely when opening and closing their eyes. The observation indexes were: motion trajectory length (mm) and motion area (mm^2^).

#### Sample Size Estimation

Based on previous clinical experience, we estimate that the total effective rate of the main indicators in the control group is 50% and we expect that the total effective rate in the intervention group can reach 80%. The sample was estimated using G^*^Power 3.1 software. A two-sided test was conducted using a rank sum test model, setting the effective rates for the intervention and control groups at 80 and 50% respectively, setting α = 0.05 and power = 0.97, and matching the distribution ratio 1:1 to obtain a final sample size of 142, taking into account an attrition rate of 12%. Therefore, the total final sample size was determined to be 160 cases, i.e., 80 cases per group. At the end of the trial, we calculated the power value for the primary indicator and the results showed that power = 0.98, indicating that our trial results were reliable. Therefore, this further indicates that our sample size met the requirements of the trial.

### Statistical Analysis

Data were processed using SPSS software (ver. 20.0). PPS (Per Protocol Set) was used to analyze the data. Grade and binary data are expressed as examples or percentages. In addition, when both the primary and secondary indicators were continuous variables and conformed to a normal distribution, the data are expressed as the mean ± standard deviation. Tests for normality found that the BBS and MBI, MPT, FMA, static standing and sitting balance while opening and closing eyes, diaphragm thickness during QB and DB, DTF, diaphragm mobility during QB and DB were all discontinuous variables and the data are expressed as the median and interquartile intervals. The Wilcoxon sign-rank test was used to compare pre- and post-treatments within groups and the Mann-Whitney test to compare pre- and post-treatment differences between groups. The significance level was set at *P* < 0.05.

## Results

### Description of Baseline Data

Table 1 is a schematic illustration of the experimental procedures. A total of 160 patients completed the study without any significant adverse effects. No significant differences in the distribution of gender, age, duration of stroke, type of stroke, BBS and MBI, MPT, FMA, diaphragm thickness during QB and DB, DTF, diaphragm mobility during QB and DB, static standing and sitting balance while patients opened and closed their eyes at baseline among the 2 groups were found (*P* > 0.05, see Table 1).

**Table 1:** General characteristics of the two groups by randomization assignment.

**Characteristic**	**Intervention (*n* = 80)**	**Control (*n* = 80)**
**Demographics**		
Age (years)	65.44 ± 9.16	62.80 ± 11.18
Gender—male : female- *n* (%)	56/24 (70.0/30.0)	64/16 (80.0/20.0)
Side of hemiplegia—left: right-n (%)	35/45 (43.7/56.3)	39/41 (48.7/51.3)
Duration of stroke (days)	67.04 ± 47.86	76.88 ± 61.63
Type of stroke–cerebral infarction: cerebral hemorrhage-n (%)	65/15 (81.2/18.8)	61/19 (76.2/23.8)

*In addition to age, onset time and other data, mean ± SD or n (%) was used, while gender, stroke type, hemiplegic side and other dichotomous data were represented by examples and percentage. The data of other observation indexes are normally distributed and expressed by the median and quartile spacing*.

### Comparison of Functional Indicators Such as BBS, MPT, MBI, and FMA

Compared to the CG, the change values of the BBS were (10.55 ± 3.78 vs. 9.06 ± 5.40, respectively, *P* = 0.039), MPT were (5.41 ± 4.70 vs. 5.89 ± 5.24, *P* = 0.001) and MBI (12.88 ± 6.45 vs. 10.00 ± 4.84, *P* = 0.001) were significantly improved in the IG (*P* < 0.05). However, there was no significant difference in the values of FMA between the 2 groups (2.39 ± 1.47 vs. 1.61 ± 0.87, *P* = 0.69) (see Table 2).

**Table 2:** A comparison of BBS, MPT, MBI, and FMA.

	**Intervention**		**Control**		**T**_**1**_ **-T**_**0**_		***P*-value**
	**T_**0**_**	**T_**1**_**	**T_**0**_**	**T_**1**_**	**Intervention**	**Control**	
BBS^a^	32 (28–37)	43 (38–49)	32 (27–38)	43 (36–47)	10.55 ± 3.78	9.06 ± 5.40	0.039
MPT^a^	6.3 (4.5–9.3)	8.7 (7.03–11.15)	6.70 (5.12–8.31)	8.0 (6.7–9.84)	5.41 ± 4.70	5.89 ± 5.24	0.001
MBI^a^	60 (55–75)	75 (70–84)	65 (55–75)	75 (65–85)	12.88 ± 6.45	10.00 ± 4.84	0.003
FMA	57 (38–83)	65 (44–89)	51 (39–81)	57 (44–85)	2.39 ± 1.47	1.61 ± 0.87	0.69

*Continuous variables are expressed using median and interquartile spacing. T_0_, baseline data; T_1_, data after 2 weeks of intervention; T_1_-T_0_ means the difference before and after intervention*.

a *Higher values indicate more favorable, the difference before and after intervention; P, Comparison of changes before and after treatment between the two groups; BBS, berg balance scale; MPT, maximum phonation time; MBI, modified Barthel index; FMA, fugl-meyer assessment*.

### Comparison of Diaphragm Function Indicators

Changes in values of diaphragmatic thickness during QB (−0.003 ± 0.022 vs. −0.003 ± 0.021, *P* = 0.96), and diaphragmatic thickness during DB (−0.007 ± 0.023 vs. −0.0004 ± 0.019, *P* = 0.07), and the DTF (0.059 ± 0.023 vs. 0.023 ± 0.22, P = 0.65) were not significantly improved between the 2 groups. Again, as can be seen in Figure 4, there was no significant trend in diaphragm thickness in either group during QB and DB. Compared to CG, there was a significant improvement in the values of diaphragmatic mobility during QB (0.54 ± 0.73 vs. 0.33 ± 0.40, *P* = 0.01) and DB (0.99 ± 1.32 vs. 0.52 ± 0.77, *P* = 0.003) in IG (see Table 3). As can be seen in Figure 4, both groups showed a significant change in increased diaphragmatic mobility during QB and DB.

**Table 3:** A comparison of diaphragm function.

	**Intervention**		**Control**		**T**_**1**_ **-T**_**0**_		***P*-value**
	**T_**0**_**	**T_**1**_**	**T_**0**_**	**T_**1**_**	**Intervention**	**Control**	
**Diaphragm thickness**							
Diaphragm thickness during QB^a^	0.22 (0.21–0.23)	0.22 (0.19–0.23)	0.22 (0.21–0.22)	0.21 (0.19–0.22)	−0.003 ± 0.022	−0.003 ± 0.021	0.96
Diaphragm thickness during DB^a^	0.37 (0.36–0.38)	0.38 (0.36–0.39)	0.37 (0.36–0.38)	0.37 (0.36–0.38)	−0.007 ± 0.026	−0.0004 ± 0.019	0.07
DTF^a^	0.71 (0.57–0.77)	0.73 (0.65–0.86)	0.68 (0.64–0.81)	0.73 (0.64–0.89)	0.059 ± 0.023	0.033 ± 0.22	0.65
**Diaphragm mobility**							
Diaphragm mobility during QB^a^	1.25 (1.02–1.61)	1.78 (1.38–2.29)	1.2 (0.98–1.56)	1.51 (1.22–1.89)	0.54 ± 0.73	0.33 ± 0.40	0.01
Diaphragm mobility during DB^a^	4.89 (4.08–5.93)	6.10 (5.21–6.76)	4.75 (3.68–5.63)	5.15 (4.21–6.44)	0.99 ± 1.32	0.52 ± 0.77	0.003

*Continuous variables are expressed using median and interquartile spacing*.

a *Higher values indicate more favorable; T_0_, baseline data; T_1_, data after 2 weeks of intervention; T_1_-T_0_, the difference before and after intervention; P, Comparison of changes before and after treatment between the two groups; QB, quiet breath; DB, deep breath; DTF (diaphragm thickness fraction) = end-inspiratory thickness—end-expiratory thickness/end-expiratory thickness × 100%*.

### Comparison of Static Balance Ability

Compared to CG, the results of static balance ability tests in the standing position with patient eyes open revealed that the values of COP trajectory (−108.34 ± 108.60 vs. −89.00 ± 140.11, *P* = 0.034) and area (−143.79 ± 431.55 vs. −93.29 ± 223.15, *P* = 0.015) were significantly improved in IG. In the seated position with patient eyes open the values of COP trajectory (−19.95 ± 23.35 vs. −12.83 ± 26.64, *P* = 0.001) and area (−15.83 ± 9.61 vs. −11.29 ± 9.17, *P* = 0.002) were significantly improved in the IG. However, there was no significant difference in COP trajectory (−110.96 ± 146.26 vs. −98.74 ± 153.48, *P* = 0.38) and area (−110.08 ± 267.91 vs. −201.36 ± 411.28, *P* = 0.54) in the sitting static state with patients eyes closed between the 2 groups, and there was no significant difference in COP trajectory (−7.39 ± 18.64 vs. −16.49 ± 41.18, *P* = 0.26)and area (−9.06 ± 8.94 vs. −9.23 ± 13.18, *P* = 0.76) in the standing static state with patients eyes closed between the 2 groups (see Table 4).

**Table 4:** A comparison of static balance ability.

	**Intervention**		**Control**		**T**_**1**_ **-T**_**0**_		***P*-value**
	**T_**0**_**	**T_**1**_**	**T_**0**_**	**T_**1**_**	**Intervention**	**Control**	
**Static open eye standing balance test**							
COP trajectory in the stand^b^	370 (276–468)	270 (202–317)	336 (258–443)	274 (232–330)	−108.34 ± 108.60	−89.00 ± 140.11	0.034
COP area in the stand^b^	279 (224–390)	185 (143–279)	273 (196–380)	216 (146–295)	−143.79 ± 431.55	−93.29 ± 223.15	0.015
**Static closed standing balance test**							
COP trajectory in the stand^b^	485 (391–622)	381 (286–480)	458 (392–576)	387 (316–475)	−110.96 ± 146.26	−98.74 ± 153.48	0.38
COP area in the stand^b^	408 (317–585)	330 (234–432)	396 (285–571)	320 (234–380)	−110.08 ± 267.91	−201.36 ± 411.28	0.54
**Static open eye sitting balance test**							
COP trajectory in the seat^b^	75 (68–84)	51 (43–61)	75 (64–89)	65 (54–75)	−19.95 ± 23.35	−12.83 ± 26.64	0.001
COP area in the seat^b^	41 (32–47)	21 (16–32)	34 (27–47)	24 (15–34)	−15.83 ± 9.61	−11.29 ± 9.17	0.002
**Static closed eye sitting balance test**							
COP trajectory in the seat^b^	87 (67–99)	77 (66–89)	86 (66–116)	80 (63–91)	−7.39 ± 18.64	−16.49 ± 41.18	0.26
COP area in the seat^b^	23 (14–43)	14 (7–31)	19 (14–38)	14 (10–27)	−9.06 ± 8.94	−9.23 ± 13.18	0.76

*Continuous variables are expressed using median and interquartile spacing*.

b *Lower values indicate more favorable; T_0_, baseline data; T_1_, data after 2 weeks of intervention; T_1_-T_0_, the difference before and after intervention; P, Comparison of changes before and after treatment between two groups; COP, center of pressure*.

## Discussion

The results of the present study confirmed that compared with traditional core stability and conventional rehabilitation training, short-term LQG combined with conventional rehabilitation training can significantly improve the balance functions of stroke patients. This approach significantly improved diaphragm functions and respiratory energy, resulting in a significant improvement in balance, and was beneficial for the quality of daily life of patients.

In the present study, the BBS and PK-254P were used to evaluate the dynamic balance ability of the patients in various functional activities, as well as the sitting and standing static balance abilities under the condition of open and closed eyes. The data on dynamic balance by PK-254P could not be obtained, because the enrolled patients did not have the ability to complete the procedure. We found that LQG had a better effect on static balance ability at the time of eye opening and the BBS compared to CST. CST is one of the most widely used methods to improve balance function, such as back curls and bridge exercises, both of which are fast and intense exercises that may be more likely to activate superficial core muscles, but not necessarily deep core muscles, and can easily aggravate spasm leading to motor mode solidification. There is an idea that the main respiratory muscles, which are also the deep core muscles, are important for balance. Therefore, effective training of the main respiratory muscle groups can improve balance, which has been supported by many studies (39, 40). Lee's study showed that after breathing training, the diaphragm and transverse abdominis muscles were effectively activated in stroke patients, leading to a significant improvement in static seated balance, dynamic seated balance (8, 41). LQG is a special breathing training method, and its soft, even, slow and long breathing pronunciation is conducive to the activation of deep core muscle. A previous study by our research team showed that LQG had a better effect on trunk control in stroke patients in comparison with conventional breathing training (42). Meanwhile, in the training process of LQG, the body should be completely relaxed with movements being extended, slow, gentle and as graceful as “flowing water and clouds,” which avoids the exacerbation of spasticity. LQG also emphasizes breathing and vocalization, accompanied by flexion, extension, lift, abduction and other movements of the upper limbs, as well as moving the center of gravity up and down and rotation of the trunk. This type of movement may be more suitable for a stroke patient to practice, gradually adjusting their center of gravity and improving postural control. The features of LGQ mentioned above should all contribute to the improvement of balance. However, we did not find that the LQG had a better effect on the static balance ability when the eyes were closed, which is significantly related to proprioception, so a longer recovery cycle may be required to achieve better results (43).

Liuzijue is a set of Qigong exercises for health and fitness, with breathing as the main body and simple guiding movements accompanying the breathing program. Our previous study showed that after 3-week intervention, LQG could effectively improve patients maximum inspiratory and maximum expiratory pressures, compared to conventional respiratory training (42). In our study, MPT was used as a general estimate to evaluate respiratory ability, which was originally considered to be an objective indicator of the efficiency of the respiratory mechanism during phonation. Yorkston et al. found that MPT more accurately reflects the maximum respiratory capacity rather than breathing support for speech (44). Before treatment, the MPT of the intervention and control groups were 6.3 and 6.7, respectively, which were significantly lower than the standard of healthy people, indicating that the respiratory ability of stroke patients was weakened. After treatment, LQG significantly improved the MPT of stroke patients compared with conventional core stability training. By extension, the result reflects a better improvement of respiration capacities. LQG should be conducted in a loose, relaxed, slow and gentle way to maintain even and prolonged breathing and pronouncing. The diaphragm, transverse abdominis and pelvic floor muscles are mainly composed of slow skeletal muscle fibers, which are more easily activated by low load and slow frequency breathing training, which is in line with the characteristics of LQG. In addition, the respiratory system and the vocal system are naturally coupled processes, so synchronized training of the two is conducive to the completion of stable and soft breathing. It is worth noting that LQG pays more attention to the training of exhalation ability, which is more suitable for stroke patients who present with a more pronounced decline in expiratory function (45). The LQG is employed to emphasize the adverse abdominal breathing method, which also contributes to respiratory functions (10–18, 20, 21, 23–29, 32–38, 42, 44–46). Therefore, LQG can improve the respiratory function of patients after stroke, and the respiratory function is beneficial to the improvement of balance ability.

Previous studies have shown that patients in the acute stage of stroke have impaired diaphragmatic function (47). About 40% of stroke patients experience a reduced diaphragmatic range of motion (48). Our study found that calm diaphragm mobility during quiet breathing and deep breathing in the intervention group produced better results than in the control group under b-ultrasound. The reason may the repeated slow deep inhalation and exhalation of LQG, as well as the expansion of the upper thoracic cage by the accompanying guidance exercise, which is conducive to increased diaphragmatic mobility. Improved mobility of the diaphragm has been shown to be beneficial for balance (49). However, our study found no improvement in diaphragm thickness, which may be related to the short intervention period.

The study demonstrated that LQG could significantly improve the MBI of stroke patients and the effect was better than that achieved by core stability training. The reason may be that as the balance functions of stroke patients were significantly improved, their MBI was also improved (50). This study confirmed that the improvement of motor functions in stroke patients could not be improved by LQG, and the reason may be related to the brief hospitalization period and short intervention time.

## Limitations

Considering that this study was a preliminary study on the influence of LQG on balance function disorders after stroke, the treatment period was only 2 weeks, to ensure a more controlled training quality and to reduce the influence of other factors. In addition, due to the limited medical resources, it is unlikely that long-term clinical trials will be conducted in inpatient units. In the future, study protocols should be designed for implementation in in community hospitals and rehabilitation facilities to ensure longer term and more reliable outcomes for stroke patients.

## Conclusion

Compared with core stability and conventional rehabilitation training, short-term LQG combined with conventional rehabilitation training significantly improved the balance functions of stroke patients. It also significantly improved diaphragm functions and respiratory energy, resulting in a significant improvement in balance functions, and was beneficial to the quality of daily life of stroke patients.

## Data Availability Statement

The original contributions presented in the study are included in the article/Supplementary Material, further inquiries can be directed to the corresponding author/s.

## Ethics Statement

The studies involving human participants were reviewed and approved by Ethics Committee of Shanghai Xuhui Central Hospital. The patients/participants provided their written informed consent to participate in this study. Written informed consent was obtained from the individual (s) for the publication of any potentially identifiable images or data included in this article.

## Author Contributions

YZ, CW, JY, LQ, YX, and SD: study concept and design and drafting of the manuscript. LQ, JY, and YX: acquisition and evaluation of data. CW, YZ, and YX: analysis of data. JW and ZY: statistical analysis. YY, SD, CW, LY, YX, and YW: case assignment. JY and WN: core stability training. All authors revised the manuscript for important intellectual content. All authors contributed to the article and approved the submitted version.

## Funding

The research was funded by the Key Specialty of Neurological Rehabilitation of Shanghai Disabled Person's Federation in 2015 and the TCM Project of Xuhui District in 2017 (No. SHXH201726) and Shanghai Health Care Commission Chinese Medicine Research Project - The effect of “Liuzijue” on trunk control and respiratory muscle function in patients with early stroke recovery compared with conventional breathing training (No. 2020LZ005).

## Conflict of Interest

The authors declare that the research was conducted in the absence of any commercial or financial relationships that could be construed as a potential conflict of interest.

## Publisher's Note

All claims expressed in this article are solely those of the authors and do not necessarily represent those of their affiliated organizations, or those of the publisher, the editors and the reviewers. Any product that may be evaluated in this article, or claim that may be made by its manufacturer, is not guaranteed or endorsed by the publisher.

## Abbreviations

BBS: Berg Balance Scale
CG: Control Group
COP: Center of Pressure
COPD: Chronic Obstructive Pulmonary Disease
CST: Core Stability Training
DB: Deep Breath
DTF: Diaphragm Thickness Fraction = end-inspiratory thickness—end-expiratory thickness/end-expiratory thickness × 100%
FMA: Fugl-Meyer Assessment
IG: Intervention Group
LQG: Liuzijue Qigong
ITT: Intention to treat
MBI: Modified Barthel Index
MMSE: Mini-Mental State Examination
MPT: Maximum Phonation Time
QB: Quiet Breath
RCT: Randomized Controlled Trial
TCM: Traditional Chinese Medicine.
